# Less efficacy with alternating regimen as adjuvant chemotherapy in stage II node-positive breast cancer: results at 8 years (Pronacam 85).

**DOI:** 10.1038/bjc.1997.423

**Published:** 1997

**Authors:** R. Chacon, L. Romero AcuÃ±a, C. Blajman, C. Galvez, M. Bruno, A. Romero, G. Chiessa, M. Bader, R. Schwan, C. Albera, M. T. Santarelli, F. Sousa MartÃ­nez, J. Nadal, M. Viniegra

## Abstract

A randomized trial to compare adjuvant treatment with an alternating regimen with conventional chemotherapy was performed. A total of 589 node-positive patients were included and stratified according to number of positive nodes (N1-3 and N > 4) and menopausal status. Premenopausal N1-3 patients were randomized to cyclophosphamide, methotrexate and fluorouracil (CMF) or CMF/4'-epirubicin, cyclophosphamide (EC), post-menopausal N1-3 patients to fluorouracil, 4 epirubicin, cyclophosphamide (FEC) or CMF/EC and pre- and post-menopausal patients with N > or = 4 to fluorouracil, 4' epirubicin, cyclophosphamide, methotrexate, prednisone (FECMP) or CMF/EC. In premenopausal patients, CMF was superior to CMF/EC in terms of disease-free survival (DFS) (65% vs 45%, P = 0.0149) and survival (72.3% vs 50.2%, P = 0.0220) whereas, for N > or = 4 patients, differences between FECMP and CMF/EC did not achieve statistical significance (DFS 35% vs 26.2%; survival 50% vs 38.1%, P = NS). For post-menopausal patients, FEC was superior to CMF/EC in DFS (58.6% vs 36.8%, P = 0.0215) and survival (66.2% vs 46%, P = 0.0155). In post-menopausal patients with N > 4, differences favouring CMF/EC were significant in DFS (40.4% vs 22%, P = 0.0371) but not in survival (47.4% vs 32.2%, P = 0.1185). Alternating regimens did not offer better results in premenopausal and post-menopausal N1-3 patients. Results regarding post-menopausal N > 4 women require further confirmation.


British Journal of Cancer (1997) 76(4), 545–550
© 1997 Cancer Research Campaign

### Less efficacy with alternating regimen as adjuvant chemotherapy in stage II node-positive breast cancer: results at 8 years (Pronacam 85)

**R Chacon, L Romero Acuña, C Blajman, C Galvez, M Bruno, A Romero, G Chiessa, M Bader, R Schwan, C Albera, MT Santarelli, F Sousa Martínez, J Nadal, M Viniegra and cooperating investigators**

Cramer 1180 – (1426), Buenos Aires, Argentina

**Summary** A randomized trial to compare adjuvant treatment with an alternating regimen with conventional chemotherapy was performed. A total of 589 node-positive patients were included and stratified according to number of positive nodes (N1–3 and N > 4) and menopausal status. Premenopausal N1–3 patients were randomized to cyclophosphamide, methotrexate and fluorouracil (CMF) or CMF/4′-epirubicin, cyclophosphamide (EC), post-menopausal N1–3 patients to fluorouracil, 4 epirubicin, cyclophosphamide (FEC) or CMF/EC and pre- and post-menopausal patients with N ≥ 4 to fluorouracil, 4′ epirubicin, cyclophosphamide, methotrexate, prednisone (FECMP) or CMF/EC. In premenopausal patients, CMF was superior to CMF/EC in terms of disease-free survival (DFS) (65% vs 45%, $P = 0.0149$) and survival (72.3% vs 50.2%, $P = 0.0220$) whereas, for N ≥ 4 patients, differences between FECMP and CMF/EC did not achieve statistical significance (DFS 35% vs 26.2%; survival 50% vs 38.1%, $P$ = NS). For post-menopausal patients, FEC was superior to CMF/EC in DFS (58.6% vs 36.8%, $P = 0.0215$) and survival (66.2% vs 46%, $P = 0.0155$). In post-menopausal patients with $N > 4$, differences favouring CMF/EC were significant in DFS (40.4% vs 22%, $P = 0.0371$) but not in survival (47.4% vs 32.2%, $P = 0.1185$). Alternating regimens did not offer better results in premenopausal and post-menopausal N1–3 patients. Results regarding post-menopausal N > 4 women require further confirmation.

**Keywords**: breast cancer; adjuvant chemotherapy; alternating regimen; randomized trial; 4′-epirubicin

Adjuvant chemotherapy reduces the risk of recurrence and death in almost all prognostic groups of women with breast cancer (EBCTCG, 1992). However, overall outcomes remain to be improved, especially for node-positive patients. Attempts to achieve better survival rates include the administration of new agents, dose intensification and strategies to overcome or prevent drug resistance. Goldie and Coldman's hypothesis states that drug-resistant clones could emerge from mutations produced before or early during chemotherapy administration (Goldie and Coldman, 1979). This model emphasizes the importance of applying as many effective drugs as possible in the shortest time interval. Alternating non-cross-resistant regimens were tested in several clinical models but, except for meclorethamine oncorin-procarbanin-prednisone-adriamycin bleomycin rinblastine DTIC (MOPP-ABVD) vs MOPP in advanced Hodgkin's disease (Bonadonna et al, 1986), their superiority to standard treatment has not been demonstrated.

This trial was designed during the early 1980s to compare 4′ epirubicin standard regimens with the alternation of cyclophosphamide, methotrexate, fluorouracil/4′ epirubicin, cyclophosphamide (CMF/EC) in node-positive women with breast cancer. Although the CMF regimen is considered to be standard treatment for premenopausal patients with one to three positive axillary nodes, outcomes for patients with more than four nodes, as well as for post-menopausal women, are still suboptimal. Consequently, anthracycline-based schemes were used in these subsets. Likewise, the combination of 4′ epirubicin and cyclophosphamide has been chosen because of its efficacy in advanced disease and the theoretical lack of complete cross-resistance with the CMF regimen.

*Received 8 August 1996*
*Revised 21 February 1997*
*Accepted 26 February 1997*

*Correspondence to:* M Viniegra, Clinical Oncology, Instituto Alexander Fleming, Cramer 1180 – (1426), Buenos Aires, Argentina

### PATIENTS AND METHODS

### Patients

Between July 1985 and July 1987, 589 consecutive women with histologically confirmed axillary node-positive breast cancer were included in this trial. Twenty-one patients were removed from the study because of major violations of inclusion criteria, treatment administration and lack of adequate follow-up data. Surgical procedures included modified radical mastectomy, quadrantectomy or tumorectomy (with uninvolved margins) plus axillary node dissection.

Patients were excluded from the study if they had clinical evidence of metastasis, were aged > 75 years or had a documented history of previous cancer (except surgically treated basal cell carcinoma of the skin or early cervical carcinoma) or any systemic condition precluding proper administration of chemotherapy. Histological analyses of more than ten axillary lymph nodes were required. Patients must have been included within 6 weeks from surgery.

Patients' characteristics (Tables 1 and 2) were homogeneously distributed across treatment arms. During the inclusion period, the technology for hormonal receptor assays was not available in many centres throughout the country, and thus receptor status was unknown for most patients (more than 70%). The high frequency of T2 tumours (80%) and patients undergoing mastectomy (70%) is also noteworthy.

**Table 1** Premenopausal patients' characteristics in relation to number of positive nodes (1–3 or ≥ 4) and treatment regimens (CMF, CMF/EC or FECMP)

| | 1–3 | | ≥ 4 | |
|---|---|---|---|---|
| | CMF | CMF/EC | CMF/EC | FECMP |
| *n* (Randomized) | 61 | 58 | 67 | 64 |
| Removed (%) | 1 (1.6) | 2 (3.4) | 3 (4.4) | 3 (4.7) |
| *n* | 60 | 56 | 64 | 61 |
| Age (years) | 40 | 42 | 42 | 42 |
| (Range) | (24–53) | (23–52) | (29–54) | (26–52) |
| Type of surgery | | | | |
| Conservative | 19 | 16 | 22 | 15 |
| Mastectomy | 41 | 40 | 42 | 46 |
| Pathological tumour size | | | | |
| 0–2 cm | 13 | 25 | 15 | 12 |
| 2–5 cm | 46 | 31 | 48 | 45 |
| Not specified | 1 | 0 | 1 | 4 |
| Histological type (*n*) | | | | |
| Invasive ductal carcinoma | 47 | 49 | 53 | 53 |
| Invasive lobular carcinoma | 8 | 3 | 9 | 7 |
| Other | 5 | 4 | 2 | 1 |

**Table 2** Post-menopausal patients' characteristics in relation to number of positive nodes (1–3 or ≥ 4) and treatment regimens (FEC, CMF/EC or FECMP)

| | 1–3 | | ≥ 4 | |
|---|---|---|---|---|
| | FEC | CMF/EC | CMF/EC | FECMP |
| *n* (Randomized) | 87 | 78 | 84 | 90 |
| Removed (%) | 2 (2.2) | 5 (6.4) | 2 (2.3) | 3 (3.3) |
| *n* | 85 | 73 | 82 | 87 |
| Age (years) | 58 | 57 | 58.5 | 60 |
| (Range) | (45–72) | (43–70) | (42–70) | (44–71) |
| Type of surgery | | | | |
| Conservative | 32 | 21 | 18 | 21 |
| Mastectomy | 53 | 52 | 64 | 66 |
| Pathological tumour size | | | | |
| 0–2 cm | 18 | 24 | 17 | 16 |
| 2–5 cm | 67 | 49 | 64 | 70 |
| Not specified | 0 | 0 | 1 | 1 |
| Histological type (*n*) | | | | |
| Invasive ductal carcinoma | 77 | 59 | 73 | 69 |
| Invasive lobular carcinoma | 3 | 6 | 5 | 11 |
| Other | 5 | 8 | 4 | 7 |

### Treatment regimens

Patients were randomly assigned to one of two regimens according to their menopausal status and number of positive axillary nodes (Figure 1). Post-menopausal status was defined by the absence of menses during the last year. Treatment regimens are presented in Table 3. Radiation therapy was administered to patients with conservative surgery during or after chemotherapy.

No dose reduction was allowed. For patients with granulocyte counts less than 1500 $mm^{-3}$ or platelet counts less than 100 000 $mm^{-3}$, chemotherapy was delayed for 1 or 2 weeks. If no haematological recovery was observed at that time, the patient was removed from study. Toxicity was recorded according to the WHO toxicity criteria.

This protocol was approved by the human investigation committees of the participating institutions.

**Figure 1** Randomization according to menopausal status and number of positive lymph nodes

### Statistical analysis

Disease-free survival (DFS; time of first adverse event) is defined as the time to first occurrence of progressive disease or death from any cause or to last follow-up, measured from time to entry to the protocol. Survival is defined as the time to death from any cause or to last follow-up, measured from time to entry to the protocol. Differences in the distribution of patients' characteristics across menopausal status were assessed using the exact test for contingency tables. Disease-free survival and survival distribution were estimated using the product limit method of Kaplan and Meier (Kaplan and Meier, 1958). The statistical significance of differences observed in the distribution of time to events was assessed using the log-rank test (Peto et al, 1977). All *P*-values reported are two sided.

### RESULTS

### Disease-free survival and survival (Table 5)

### *Premenopausal patients*

Comparison between CMF and CMF/EC in patients with 1–3 positive nodes disclosed statistically significant differences in DFS, in favour of CMF (log-rank test $P = 0.015$), with estimated 8-years DFS being 65% with CMF (95% CI 51–76%) vs 45% (95% CI 32–58%) with CMF/EC. These differences were also apparent for survival ($P = 0.0220$), with estimated 8-year survival of 72.3% (95% CI 59–82%) vs 50.2% (36–66%) with CMF and CMF/EC respectively. Figure 2A and B discloses survival curves for both treatment arms. A median DFS of 49 months for patients in the CMF/EC arm was observed, whereas the median for CMF patients has not yet been reached.

On the other hand, in patients with more than four positive lymph nodes, CMF/EC has not produced better outcomes than FECMP neither in DFS (log-rank test $P = 0.3160$) with estimated 8-year DFS of 26.2% (95% CI 14–40%) with CMF/EC and 35% with FECMP (95% CI 21–49%) (Figure 2C) nor in survival ($P = 0.2879$) with estimated 8-year survival of 38.1% (95% CI 24–52%) vs 50% (95% CI 36–62.5%) (Figure 2D). This lack of difference persisted even when data were analysed for different positive node strata (4–7 and > 8) (data not shown). Median DFS and survival in patients treated with CMF/EC were shorter than those with FECMP (60 and 83 months vs 77 and 96 months respectively).

*© Cancer Research Campaign 1997*

**Table 3** Treatment regimens

| Treatment | CMF[a] | CMF/EC[b] | FECMP[a] | FEC[a] |
|---|---|---|---|---|
| Cyclophosphamide | 600 mg m$^{-2}$ D1 | 600 mg m$^{-2}$ D1 and 21 | 400 mg m$^{-2}$ D1 | 500 mg m$^{-2}$ D1 |
| Methotrexate | 40 mg m$^{-2}$ D1 | 40 mg m$^{-2}$ D1 | 30 mg m$^{-2}$ D1 | – |
| Fluorouracil | 600 mg m$^{-2}$ D1 | 600 mg m$^{-2}$ D1 | 400 mg m$^{-2}$ D1 | 500 mg m$^{-2}$ D1 |
| 4′ Epirubicin | – | 60 mg m$^{-2}$ D21 | 60 mg m$^{-2}$ D1 | 60 mg m$^{-2}$ D1 |
| Prednisone | – | – | 40 mg m$^{-2}$ d$^{-1}$ D1 to 5 | – |

[a]CMF, FECMP and FEC were administered every 21 days for six cycles. [b]CMF/EC was administered every 42 days for three cycles. D, day.

**Table 4A** Relative dose intensity for treatment regimens

| Treatment | CMF | CMF/EC | FECMP | FEC |
|---|---|---|---|---|
| Cyclophosphamide | 1 | 1 | 0.66 | 0.83 |
| Methotrexate | 1 | 0.5 | 0.75 | 0 |
| Fluorouracil | 1 | 0.5 | 0.66 | 0.83 |
| 4′ Epirubicin | 0 | 0.5 | 1 | 1 |
| Prednisone | 0 | 0 | 1 | 0 |

**Table 4B** Received dose intensity (mg per week)

| | Premenopausal | | | | Post-menopausal | | | |
|---|---|---|---|---|---|---|---|---|
| | N1–3 | | N ≥ 4 | | N1–3 | | N ≥ 4 | |
| Treatment | CMF | CMF/EC | CMF/EC | FECMP | FEC | CMF/EC | CMF/EC | FECMP |
| Cyclophosphamide | 215.6 | 213.0 | 216.9 | 137.1 | 168.5 | 209.3 | 204.5 | 141.2 |
| Methotrexate | 14.4 | 7.1 | 7.2 | 10.3 | – | 7.0 | 6.8 | 10.6 |
| Fluorouracil | 215.6 | 106.0 | 108.4 | 137.1 | 168.5 | 104.6 | 102.3 | 141.2 |
| 4′ Epirubicin | – | 10.6 | 10.8 | 20.6 | 20.2 | 10.5 | 10.2 | 21.2 |
| Prednisone | – | – | – | 68.6 | – | – | – | 70.6 |

### *Post-menopausal patients*

In the subset with one to three positive nodes, patients randomized to FEC did better than those in the CMF/EC arm (log-rank test for DFS $P$ = 0.0215), achieving a better estimated 8-year DFS – 58.6% for FEC (95% CI 47–69%) vs 36.8% for the alternating scheme (95% CI 25–49%) (Table 5). Median DFS was 71 months for CMF/EC. Median DFS has not been reached for patients receiving FEC (Figure 3A). Differences in survival were also significant ($P$ = 0.0155), with estimated 8-year survival of 66.2% (95% CI 54–76%) for FEC vs 46% (95% CI 33–58%) CMF/EC; and the median survival for the FEC group was 25 months longer than that for the CMF/EC group (116 vs 91 months) (Figure 3B).

Regarding patients with more than four lymph nodes, unexpected differences favouring the alternating chemotherapy group were observed in DFS ($P$ = 0.0371). Estimated 8-year DFS was 40.4% (95% CI 29–51%) with CMF/EC vs 22% (95% CI 13–32%) for FECMP; whereas survival figures have reached no statistical significance ($P$ = 0.1185), with estimated 8-year survival of 47.4% (95% CI 36–58%) vs 32.2% (95% CI 22–43%) respectively (Figure 3C and D). These results are not related to distribution of prognostic factors, such as number of positive nodes or tumour size, as these variables were homogeneously distributed across treatment arms.

Major sites of first relapse of disease were bone (18.2–31%), skin (9.3–16.7%), liver (8.1–17.6%) and lung (5–16.3%) as well as multiple metastatic involvement (15–33.1%). Multiple metastatic involvement as the first manifestation of recurrence was more frequently observed in premenopausal than in post-menopausal patients (24.3% vs 16.7%). No other differences were found when the pattern of relapse was analysed in relation to menopausal status, nodal groups and chemotherapy regimens.

### Toxicity

Information about toxicity is shown in Table 6. No patient required hospitalization for toxic complications. No platelet or red cell transfusions were administered. The most frequently reported side-effects were emesis and alopecia. The percentage of courses complicated with grade 3–4 emesis was slightly higher in the anthracycline-containing regimens. The alternating regimen was not less toxic than FECMP or FEC as long as anthracycline-related alopecia was considered (76.6% for CMF/EC vs 85.1% and 80% for FEC and FECMP respectively, $P$ = 0.3634). No case of clinically overt congestive heart failure was observed.

Two treatment-related deaths were reported. The first patient developed liver failure during CMF treatment, while the second patient suffered sudden death during FEC chemotherapy. Twelve further patients died without evidence of relapse months to years after completing chemotherapy. Information about causes of death was obtained from clinical reports as autopsy was not performed in

© Cancer Research Campaign 1997 

**Figure 2** Survival curves for premenopausal patients. (**A**) Disease-free survival for patients with one to three positive lymph nodes. (**B**) Survival for patients for one to three positive lymph nodes. (**C**) Disease-free survival for patients with four or more positive lymph nodes. (**D**) Survival for patients with four or more positive lymph nodes

**Table 5** Estimated 8-year DFS and survival for different treatment groups

| | Positive nodes | *n* | Regimen | DFS (%) | 95% CI | *P* | Survival (%) | 95% CI | *P* |
|---|---|---|---|---|---|---|---|---|---|
| Premenopausal | 1–3 | 60 | CMF | 65 | 50.7–76 | 0.0149 | 72.3 | 58.8–82.1 | 0.0220 |
| | | 56 | CMF/EC | 45.1 | 31.6–57.8 | | 50.2 | 35.6–65.6 | |
| | ≥ 4 | 64 | CMF/EC | 26.2 | 14.1–40 | 0.3160 | 38.1 | 24.4–51.8 | 0.2879 |
| | | 61 | FECMP | 35 | 21.2–48.9 | | 50 | 35.7–62.5 | |
| Post-menopausal | 1–3 | 85 | FEC | 58.6 | 46.7–68.7 | 0.0215 | 66.2 | 54.2–75.8 | 0.0155 |
| | | 73 | CMF/EC | 36.8 | 24.9–48.7 | | 46 | 32.9–57.8 | |
| | ≥ 4 | 82 | CMF/EC | 40.4 | 29.3–51.3 | 0.0371 | 47.4 | 35.7–58.1 | 0.1185 |
| | | 87 | FECMP | 22 | 13.3–32.2 | | 32.2 | 22–42.8 | |

any case. Eight patients died of coronary conditions, one of stroke; there was one case of community overwhelming sepsis, one fatal gastrointestinal haemorrhage and one sudden death. No patient with coronary disease received radiotherapy to the left breast.

### Second tumours

Ten patients developed second neoplasms. In eight cases the site was the opposite breast; three of them are still disease free. One patient had diagnosis of ovarian cancer and another had lung cancer. Both patients died of disseminated disease attributable to second malignancy.

### DISCUSSION

Goldie and Coldman's (1979) hypothesis, which is challenged by this study, emphasizes the possibility of overcoming drug resistance by the administration of non-cross-resistant chemotherapeutic regimens. This assumption was based on a mathematical model as well as on experimental and early clinical observations regarding advanced Hodgkin's disease and small-cell lung cancer (Evans et al, 1987).

Our results in patients with one to three positive axillary nodes suggest that six full cycles of CMF in premenopausal or FEC in post-menopausal women are superior to the administration of CMF/EC regimen for both DFS and survival. One possible

© Cancer Research Campaign 1997

**Figure 3** Survival curves for post-menopausal patients. (**A**) Disease-free survival for patients with one to three positive lymph nodes. (**B**) Survival for patients for one to three positive lymph nodes. (**C**) Disease-free survival for patients with four or more positive lymph nodes. (**D**) Survival for patients with four or more positive lymph nodes

**Table 6** Toxicity among different treatment regimens

| | CMF | CMF/EC | FEC | FECMP |
|---|---|---|---|---|
| WBC[a] | | | | |
| Grade 3 | < 1 | < 1 | < 1 | < 1 |
| Grade 4 | 0 | 0 | 0 | < 1 |
| Platelets[a] | | | | |
| Grade 3–4 | 0 | 0 | 0 | 0 |
| Emesis[a] | | | | |
| Grade 3 | 8.3 | 9.2 | 14.8 | 11.3 |
| Grade 4 | 0 | < 1 | < 1 | < 1 |
| Alopecia[b] | | | | |
| Grade 2 | 14 | 26.6 | 6.1 | 13.8 |
| Grade 3 | 8.3 | 50 | 79 | 66.2 |
| Mucositis[a] | | | | |
| Grade 3 | < 1 | < 1 | < 1 | < 1 |
| Grade 4 | < 1 | < 1 | 0 | 0 |
| Cardiac (treatment-related sudden death) | | | 1 | |
| Non-tumoral death (no. of events) | | | | |
| Total | 2 | 6 | 3 | 3 |
| Treatment related | 1 | 0 | 1 | 0 |

[a]Percentage of courses complicated with toxicity. [b]Percentage of patients presenting this complication. WBC, white blood cell count.

explanation for this outcome is that the importance of delivering critical amounts of a drug in a given interval (dose intensity) outweighs that of more erratic exposure to agents with different mechanisms of action and resistance. Similar conclusions were drawn by other groups in early and advanced breast cancer (Spittle et al, 1987; Budzar et al, 1988; Falkson et al, 1991; Bonadonna et al, 1995). Conversely, results from the ECOG (Tormey et al, 1992) showed prolonged DFS in patients receiving alternating chemo-hormonotherapy in the adjuvant setting, although the use of hormonal manipulation obscures the interpretation of data.

© Cancer Research Campaign 1997

On the other hand, for patients with more than four positive lymph nodes, results diverge depending on menopausal status. For younger patients, a trend favouring FECMP did not reach statistical significance. This effect could reflect the actual lack of difference or type II error related to the low statistical power of the sample.

Findings regarding the superiority of CMF/EC in post-menopausal patients deserve separate consideration. Although dose intensity seems to be one of the – if not the most – important features of breast cancer regimens, the relative contribution of each drug to the final outcome is still unknown. In respect to that mentioned above, it is likely that FECMP does not fit the requirements of an adequate regimen for dose intensity purposes (see Table 4). Noteworthy are the results reported by Peters et al (1994) about the critical importance of 5-FU dose intensity. Furthermore, differences in pre- and post-menopausal tumour biology may account for the apparent superiority of a non-cross-resistant regimen as they do for the lower benefit from chemotherapy reported for older women (EBCTCG, 1992).

Recent reports have raised the concern of anthracycline-related carcinogenesis. It is noteworthy that no case of treatment-related leukaemia has been observed among these more than 500 women with a median follow-up of 8 years. Likewise no report of cumulative cardiomyopathy was registered, confirming the low frequency of such a complication in the current adjuvant setting (Chacon et al, 1992).

In summary, our results suggest that alternating regimens offer no advantage or even can compromise critical end points in pre- and post-menopausal women with one to three positive axillary nodes and premenopausal patients with more than four nodes. The benefits of CMF/EC in post-menopausal patients with more than four positive nodes require further confirmation.

### ACKNOWLEDGEMENTS

We are indebted to Dr Jose Mordoh for his opinions and advice. This study was supported by a grant from Pharmacia.

### REFERENCES

Bonadonna G, Valagussa P and Santoro A (1986) Alternating non-cross resistant combination chemotherapy or MOPP in stage IV Hodgkin's disease: a report of 8-year results. *Ann Intern Med* **104**: 739–746

Bonadonna G, Zambetti M and Valagussa P (1995) Sequential or alternating Doxorubicin and CMF regimens in breast cancer with more than three positive nodes. Ten-year results. *JAMA* **273**: 542–547

Budzar AU, Hortobagyi G, Smith T, Cau S, Marcus C, Holmes S, Ghu V, Fraschini G, Ames F and Martin R (1988) Adjuvant therapy of breast cancer with or without additional treatment with alternate drugs. *Cancer* **62**: 2098–2104

Chacon R, Galvez C, Romero Acuña L, Blajman C, Machiavelli M, Schwan R, Sousa Martinez F, Nadal J, Block J et al (1992) A clinical analysis of cardiac toxicity and other life threatening toxicities in patients receiving anthracyclines as adjuvant treatment of breast cancer. Pronacam Cooperative Group. *Proc ASCO* **11**: A111

Early Breast Cancer Trialists Cooperative Group (EBCTCG) (1992) Systemic treatment of early breast cancer by hormonal, cytotoxic and immune therapy. *Lancet* **339**: 1–15, 71–85

Evans WK, Feld R, Murray N, Willan A, Coy P, Osoba D, Shepherd F, Clark D, Levitt M, MacDonald A, Wilson K, Shelley W and Pater J (1987) Superiority of alternating non cross resistant chemotherapy in extensive small cell lung cancer. *Ann Intern Med* **107**: 451–458

Falkson G, Tormey DC, Carey P, Witte R and Flakson H (1991) Long term survival of paitents treated with combination chemotherapy for metastatic breast cancer. *Eur J Cancer* **27**: 973–977

Goldie JH and Coldman AJ (1979) A mathematical model for relating the drug sensitivity of tumors or their spontaneous mutation rate. *Cancer Treat Rep* **63**: 1727

Kaplan EL and Meier P (1958) Non parametric estimation for incomplete observation. *J Am Stat Assoc* **53**: 457–481

Peto R, Pike M and Armitage P (1977) Design and analysis of randomized clinical trials requiring prolonged observation of each patient. Analysis and examples. *Br J Cancer* **35**: 1–39

Peters WP, Petros W, Gupton C, Vreddenburgh J, Hussein A, Ross M, Rubin P, Elkordy M, Affronti M and Moore S (1994) The effect of induction chemotherapy dose intensity on complete response frequency in early metastatic breast cancer. *Proc ASCO* **13**: A150

Spittle MF, Hill BT, Ostrowski M, Macrea K, Bates I, Martin W, Nicol N and Edelstyn G (1987) A randomized, prospective, comparative multicentre trial of a single combination versus alternating combinations of antitumor drugs in advanced breast cancer. *Eur J Cancer Clin Oncol* **23**: 1155–1162

Tormey DC, Gray R, Abeloff M, Roseman D, Gilchrist K, Barylak E, Stott P and Falkson G (1992) Adjuvant therapy with a doxorubicin regimen and long term use of Tamoxifen in premenopausal breast cancer patients: an Eastern Cooperative Oncology Group Trial. *J Clin Oncol* **10**: 1848–1856

### APPENDIX

Investigators contributing to this study: F Coppola, O Gómez, C Bass, J Martinez, L Borghi, J Lebron, H Miles, M Rondinon, E Minckiewicz, C Galvez, R Chacon, G Cavarra, J Loza, F Sousa Martinez, D Levy, A Marantz, R Estevez, O Rivero, M Bruno, B Diaz, G Pascon, M Freue, A Longarte, R Villegas, G Chiessa, J Arrieta, A Larravide, C Fernandez, R Loiacomo, G Puricelli, A Hannois, G Iparraguirre, D Beloqui, C Ivulich, M Kotliar, A Shmilovich, A Allami, H Muro, H Florio, M Machiavelli, J Perez, A Romero, M Escudero, R Schwan, L Silberman, E Pejko, J Chavanne, B Nuñez, A Osuna, R Pinkevicius, E Richardet, C Lazaris, G Jarchum, R del Castillo, N Garello, V Bove, S Zunino, M Di Notto, B Nores, M Matwiejuk, M Frangioli, R La Palma, D Morgan and A Paris.

 © Cancer Research Campaign 1997


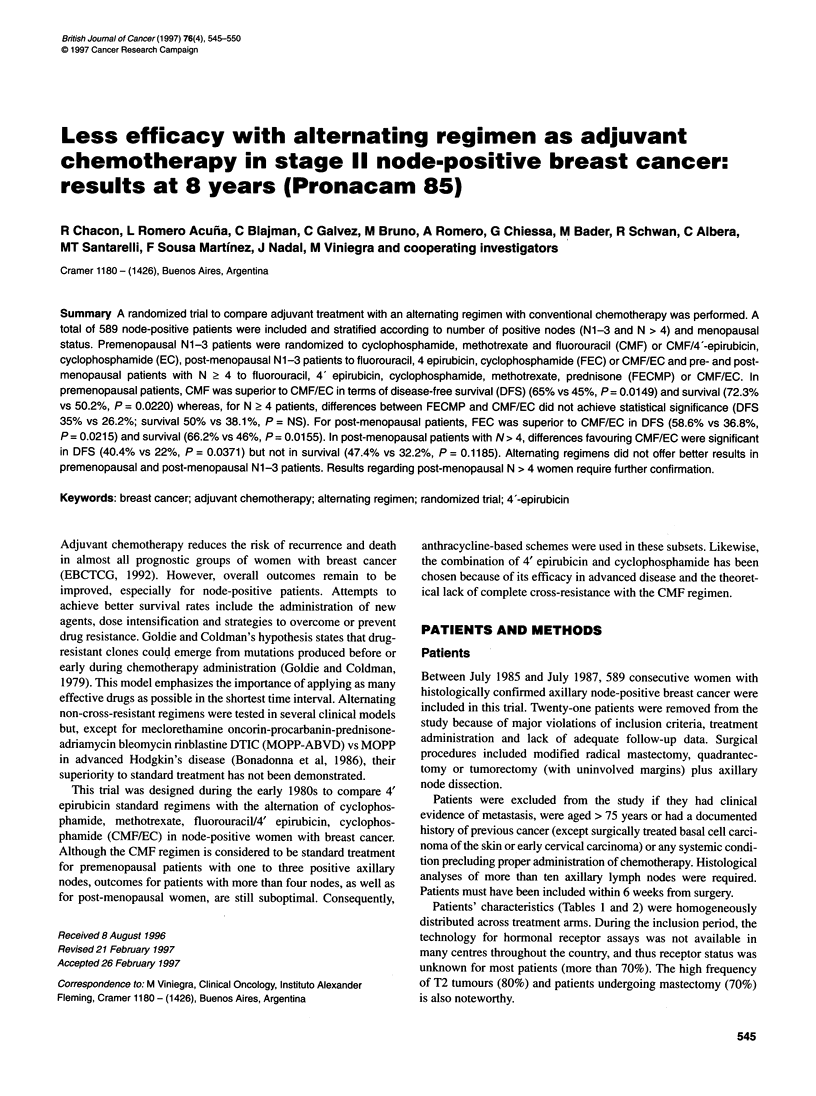

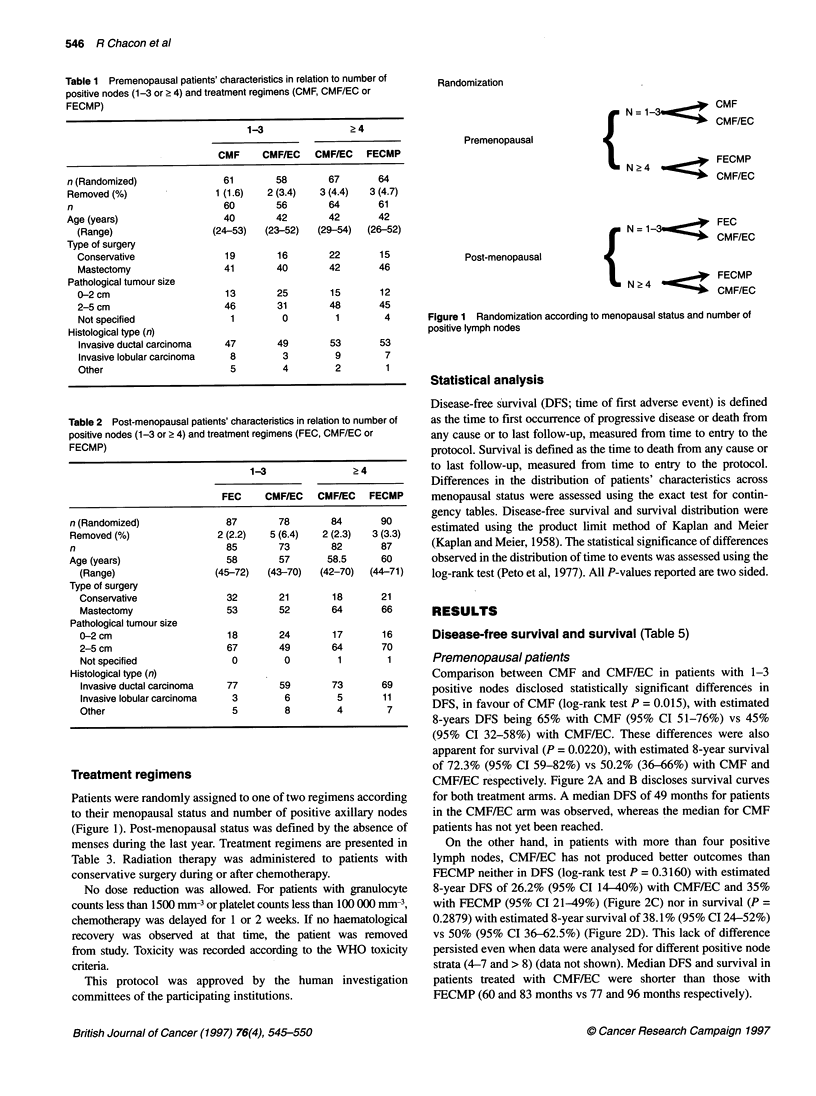

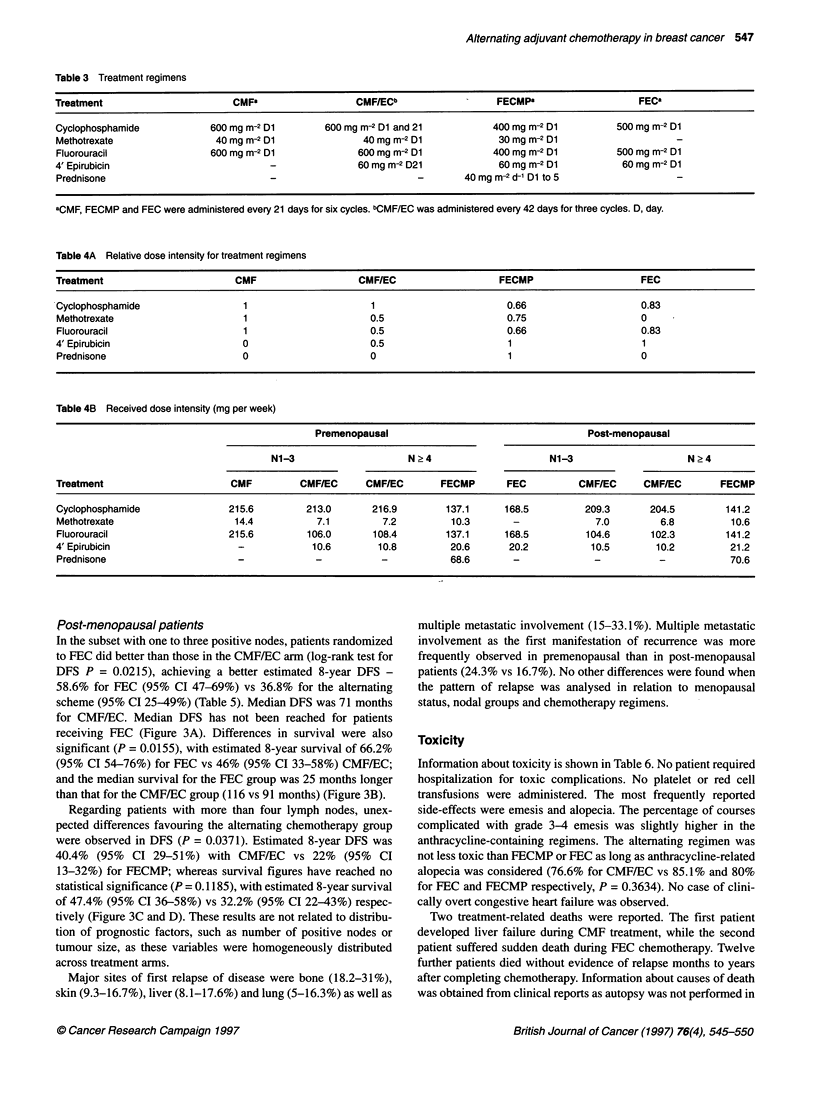

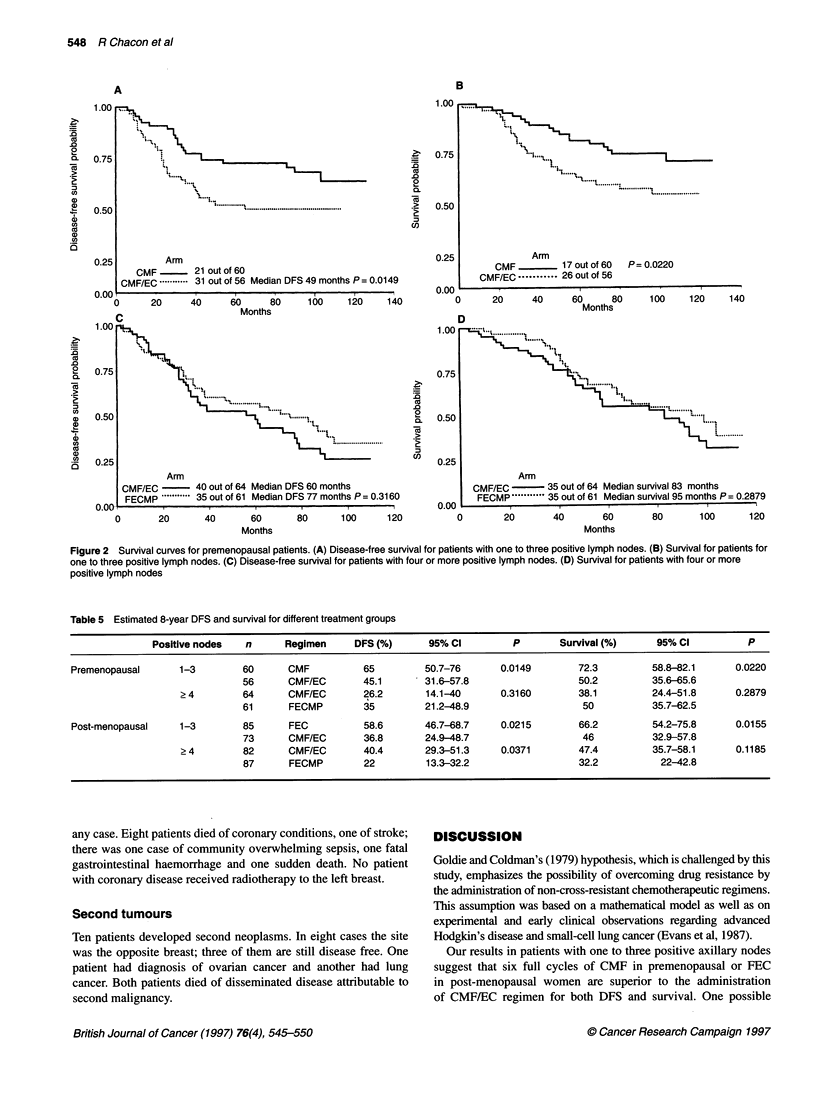

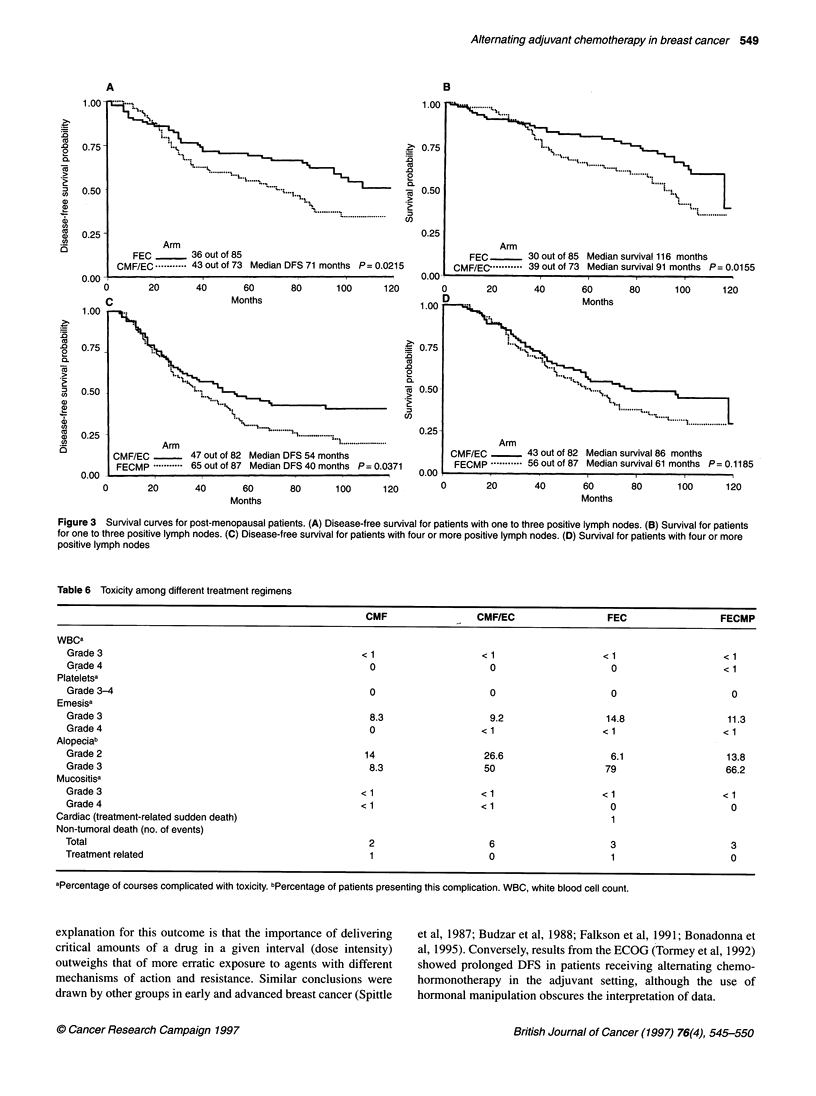

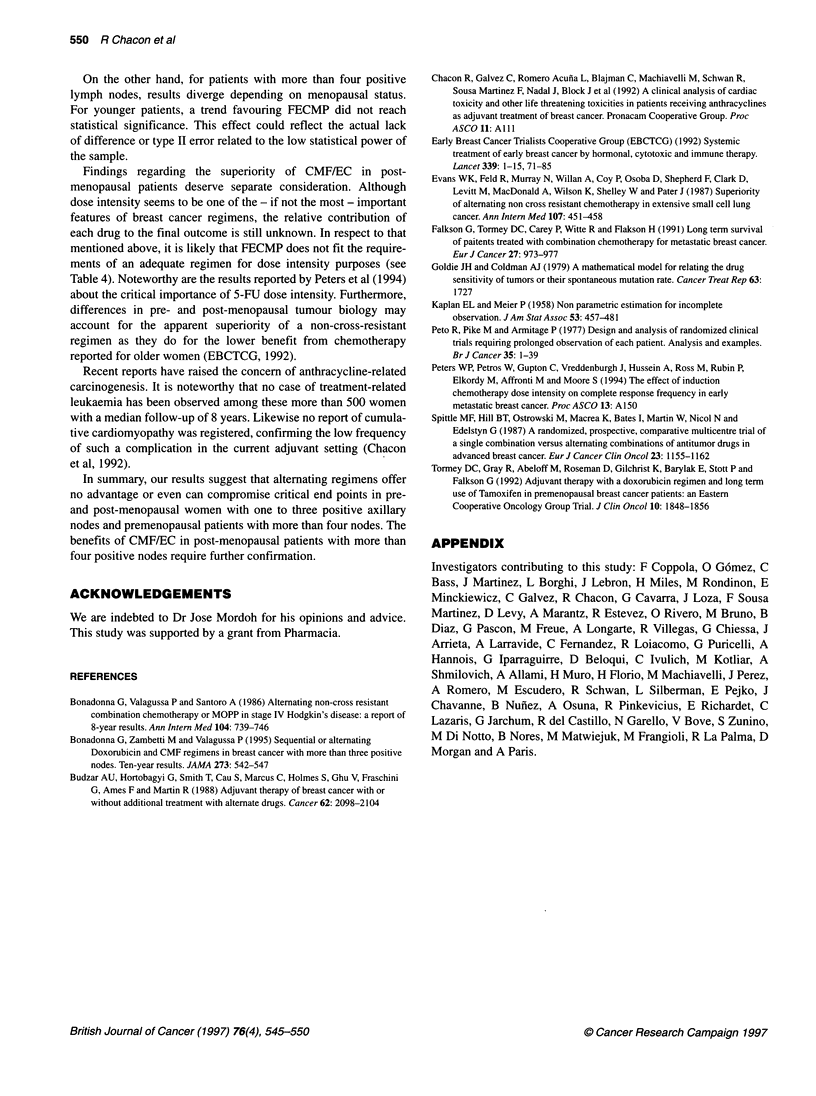


## References

[OCR_00837] Bonadonna G., Valagussa P., Santoro A. (1986). Alternating non-cross-resistant combination chemotherapy or MOPP in stage IV Hodgkin's disease. A report of 8-year results.. Ann Intern Med.

[OCR_00842] Bonadonna G., Zambetti M., Valagussa P. (1995). Sequential or alternating doxorubicin and CMF regimens in breast cancer with more than three positive nodes. Ten-year results.. JAMA.

[OCR_00847] Buzdar A. U., Hortobagyi G. N., Smith T. L., Kau S., Marcus C., Holmes F. A., Hug V., Fraschini G., Ames F. C., Martin R. G. (1988). Adjuvant therapy of breast cancer with or without additional treatment with alternate drugs.. Cancer.

[OCR_00865] Evans W. K., Feld R., Murray N., Willan A., Coy P., Osoba D., Shepherd F. A., Clark D. A., Levitt M., MacDonald A. (1987). Superiority of alternating non-cross-resistant chemotherapy in extensive small cell lung cancer. A multicenter, randomized clinical trial by the National Cancer Institute of Canada.. Ann Intern Med.

[OCR_00871] Falkson G., Tormey D. C., Carey P., Witte R., Falkson H. C. (1991). Long-term survival of patients treated with combination chemotherapy for metastatic breast cancer.. Eur J Cancer.

[OCR_00876] Goldie J. H., Coldman A. J. (1979). A mathematic model for relating the drug sensitivity of tumors to their spontaneous mutation rate.. Cancer Treat Rep.

[OCR_00885] Peto R., Pike M. C., Armitage P., Breslow N. E., Cox D. R., Howard S. V., Mantel N., McPherson K., Peto J., Smith P. G. (1977). Design and analysis of randomized clinical trials requiring prolonged observation of each patient. II. analysis and examples.. Br J Cancer.

[OCR_00897] Spittle M. F., Hill B. T., Ostrowski M. J., Macrae K. D., Bates T. D., Martin W. M., Nicol N. T., Edelstyn G. A. (1987). A randomized, prospective, comparative, multicentre trial of a single combination versus alternating combinations of antitumour drugs in advanced breast cancer.. Eur J Cancer Clin Oncol.

[OCR_00903] Tormey D. C., Gray R., Abeloff M. D., Roseman D. L., Gilchrist K. W., Barylak E. J., Stott P., Falkson G. (1992). Adjuvant therapy with a doxorubicin regimen and long-term tamoxifen in premenopausal breast cancer patients: an Eastern Cooperative Oncology Group trial.. J Clin Oncol.

